# Efficacy and safety of PD-1/PD-L1 inhibitors alone or in combination in the treatment of metastatic or advanced renal cell carcinoma: a network meta-analysis

**DOI:** 10.3389/fimmu.2025.1524497

**Published:** 2025-02-25

**Authors:** Dongli Zhang, Chong Shen, Weichuan Zhang, Haibin Chen, Jianjun Zhao

**Affiliations:** ^1^ Hebei University of Engineering School of Medicine, Handan, Hebei, China; ^2^ Second Department of Urology and Surgery, Affiliated Hospital of Hebei Engineering University, Handan, China

**Keywords:** renal cell carcinoma, PD-1/PD-L1 inhibitor, efficacy, safety, meta-analysis

## Abstract

**Background:**

This study systematically reviews the efficacy and safety of the single or combined use of programmed factor 1 (PD-1)/programmed factor 1 ligand (PD-L1) inhibitors for treating metastatic or advanced renal cell carcinoma (RCC).

**Methods:**

Relevant articles were collected for meta-analysis through searches on PubMed, Web of Science, Embase, Cochrane Library, and Clinical Trials, as well as for relevant randomized controlled experiments.

**Results:**

Based on eleven studies, the effectiveness of the experimental group was found to be significantly better than the control in terms of overall survival (OS) [R=0.74, 95%CI: 0.69~0.80, *P*<0.00001], progression-free survival (PFS) [HR=0.68, 95%CI: 0.57~0.81, *P*<0.0001], objective response rate (ORR) [RR=1.71, 95%CI: 1.39~2.12, *P*<0.00001], complete response rate (CR) [RR=2.99 95%CI: 2.34~3.83, *P*<0.0001], partial response rate (PR) [RR=1.56, 95%CI: 1.20~2.01, *P*=0.001], and disease control rate (DCR) [RR=1.13, 95%CI: 1.06~1.20, *P*<0.0001]. No statistical significance was observed between the experimental and control groups in overall adverse reactions (AEs) [RR=1.01, 95%CI: 0.98~1.04, *P*=0.598], the incidence of stage I~II adverse reactions [RR=1.02, 95%CI: 0.88~1.17, *P*=0.818], or stage III~V adverse reactions [RR=0.98, 95%CI: 0.81~1.18, *P*=0.817]. Regarding subgroup analysis, the incidence of dysphonia, rash, hypothyroidism, arthralgia, and pruritus in the experimental group was significantly higher than in the control. Compared with the control group, the incidence of diarrhea, nausea, indigestion, and fatigue in the experimental group was not statistically significant.

**Conclusion:**

A good efficacy was found in treating metastatic or advanced RCC using PD-1/PD-L1 inhibitors alone or in combination, which significantly improved and enhanced OS, PFS, ORR, CR, PR, and DCR in patients with RCC. The incidence of adverse reactions in patients was not increased, and adverse reactions were controllable. These findings indicate that the single or combined use of PD-1/PD-L1 inhibitors shows good efficacy and safety in the treatment of metastatic or advanced RCC.

## 1 Introduction

Renal cell carcinoma (RCC) is a malignancy of the urinary tract originating in the tubular epithelium, accounting for approximately 80–90% of renal malignancies (1). It is the sixth and eighth most common cancer in both men and women (2). In recent years, its morbidity has increased at a rate of 1.6% per year. A poor prognosis is more common in men (3). At present, surgery remains the main treatment option for patients with RCC. However, about 25% of patients have middle or advanced-stage RCC or distant metastasis at the time of initial diagnosis. In addition, approximately 20–50% of patients with localized renal cell carcinoma eventually develop into metastatic renal cell carcinoma (mRCC), which is insensitive to conventional chemo-radiotherapy and exhibits multi-drug-resistance (4), resulting in a survival rate of less than five years after surgery for over 80% of patients (5).

In recent years, tumor immunotherapy has offered new treatment options for mRCC or advanced renal cell carcinoma (aRCC). Programmed cell death protein 1 (PD-1) is a member of the B7-CD28 co-stimulatory receptor family and is mainly expressed in activated T lymphocytes, B lymphocytes, and monocytes, while Programmed death-ligand 1 (PD-L1) is mainly expressed in tumor cells. These two molecules are specifically combined and showed abnormally high expression in tumor tissues, which is involved in the negative regulation of human immunity. They inhibit the survival and proliferation of T immune cells and the release of killer cytokines in the local microenvironment, as well as inducing cell apoptosis, thereby preventing the immune response against tumor cells and promoting the growth of tumor cells (6). PD-1/PD-L1 inhibitors can effectively block this effect, enhance the immune function of T cells, and exert immune function on tumor cells. Additionally, PD-1/PD-L1 inhibitors, as immune sentinel monoclonal antibodies, have been widely used in treating melanoma, lung cancer, lymphoma and kidney cancer, especially in treating mRCC or aRCC (7). Studies have shown that in renal cell carcinoma, PD-1/PD-L1 alone or in combination with conventional targeted drugs can improve anti-cancer efficacy and significantly extend survival rates (8). At present, based on IMmotion 010, CheckMate 025, CheckMate 214, and KEYNOTE-426 experiments, a multicenter randomized controlled study has been performed to evaluate the efficacy and safety of the single or combined use of PD-1/PD-L1 inhibitors in the treatment of mRCC or aRCC. However, its conclusions vary.

In this study, a systematic review of the published research literature on PD-1/PD-L1 inhibitors is conducted to comprehensively evaluate the clinical efficacy and safety of PD-1/PD-L1 inhibitors in the treatment of mRCC or aRCC in an attempt to provide a rationale for the immunotherapy of mRCC or aRCC.

## 2 Materials and methods

### 2.1 General materials

#### 2.1.1 Inclusion and exclusion criteria

(i) Study subjects: patients with mRCC or aRCC that have been confirmed by histopathology and received no radiotherapy. No requirements regarding age, gender or medication history,but TNM stage required to be metastatic or locally advanced. (ii) study type: stage II or III randomized controlled trials (RCTs) that have been published (English only). (iii) interventions: in the experiment, anti-PD-1 inhibitors or anti-PD-L1 inhibitors were applied alone or in combination with other drugs (either immunological drugs, conventional chemotherapy drugs, or placebo in the case of the control group).

#### 2.1.2 Outcome index

(i) Effective index: overall survival (OS), progression-free survival (PFS), objective response rate (ORR), complete response rate (CR), partial response rate (PR), and disease control rate (DCR). (ii) safety index: incidence of treatment-related adverse reactions (AEs) and subgroup analysis (the incidence of adverse events in respiratory, digestive, and other systems).

#### 2.1.3 Exclusion criteria

(i) Duplicates, case reports, and review literature; (ii) retrospective literature; (iii) literature with abstracts only or without results; (iv) non-controlled literature; and (v) non-randomized controlled phase II and III clinical trial research.

### 2.2 Search methods for the identification of relevant studies

A detailed literature review was conducted using the following electronic databases: PubMed(https://pubmed.ncbi.nlm.nih.gov/), Web of Science(https://www.webofscience.com/wos/woscc/basic-search), Embase(https://www.embase.com/landing?status=grey, Cochrane Library(https://www.cochranelibrary.com/), and Clinical Trials(https://classic.clinicaltrials.gov/). Manual research methods were used to collect randomized controlled studies of PD-1/PD-L1 inhibitors for treating renal cell carcinoma. The research period was from the establishment of the database to September 30, 2024. Subject words and free words, search terms included: renal neoplasms, kidney cancers, renal cell carcinomas, collecting duct carcinoma of the kidney, tumor, cancer, PD-1, PD-L1, programmed cell death protein 1, programmed cell death protein 1 inhibitors, immune checkpoint inhibitor, PD-L1 inhibitors, nivolumab, pembrolizumab, lambrolizumab, atezolizumab, tecentriq, durvalumab, imfinzi, bavencio, avelumab, camrelizumab, and sintilimab.

### 2.3 Study selection and data collection

Two researchers independently screened the literature according to the inclusion and exclusion criteria, and relevant information was extracted. Line cross-checking was performed, and third-party discussions were conducted to resolve any discrepancies in extraction. During the literature screening, the title and abstracts were read first. Then, after excluding any unrelated literature, the full text was further read to determine its inclusion. Data extraction mainly included: (i) basic information of the included studies, including the study title, first author, publication journal, country, and time; (ii) baseline characteristics of the study subjects, including the number of included patient cases, histopathological type, gender and age ratio, and follow-up time; (iii) specific details of the interventions; (iv) key elements of risk of bias evaluation; and (v) the outcome indicators and outcome measures of interest, the former of which included ORR, PFS, PR, CR, OS, SD, PD, DCR, and AEs. In addition, further detailed data were collected on the International Metastatic Renal Cell Carcinoma Combined Database Hazard Score for OS, the PD-L1 expression of PRS, the IMDC score, the Memorial Sloan Kettering Cancer Centre score, and the proportion of common adverse reactions occurring in each system of the AEs.

### 2.4 Quality assessment and risk of bias

Two researchers assessed the quality of the included studies according to the Cochrane 5.3 manual, and the evaluation criteria included (i) randomization, (ii) group hiding, (iii) blinding, (iv) data integrity, (v) selective reporting, and (vi) other biases.

### 2.5 Statistical analysis

Stata15.1 software (https://www.stata.com/stata15/) was used for meta-analysis. The risk ratio (HR) and 95% confidence interval (CI) were used to determine effect sizes and evaluate the relevant degree between the experimental group and OS and PFS. The relative risk (RR) and 95% feasible interval (CI) were used as the effect size to assess the degree of association between the experimental group and ORR, PR, CR, DCR, and AEs. According to the recommendations provided by the Cochrane Handbook for Systematic Reviews of Interventions (https://training.cochrane.org/handbook/current/chapter-10#section-10-10-4-1), ‘the choice between a fixed-effect and a random-effects meta-analysis should never be made on the basis of a statistical test for heterogeneity.’ In addition, since heterogeneity is always expected for the intervention effects among multiple studies from different groups and geographical locations, a random effects model is encouraged to account for these analyses. α=0.05 was used as the test level for the meta-analysis. α=0.05 was used as the test level for the meta-analysis.

## 3 Results

### 3.1 Search results and quality assessment

**Figure 1:**
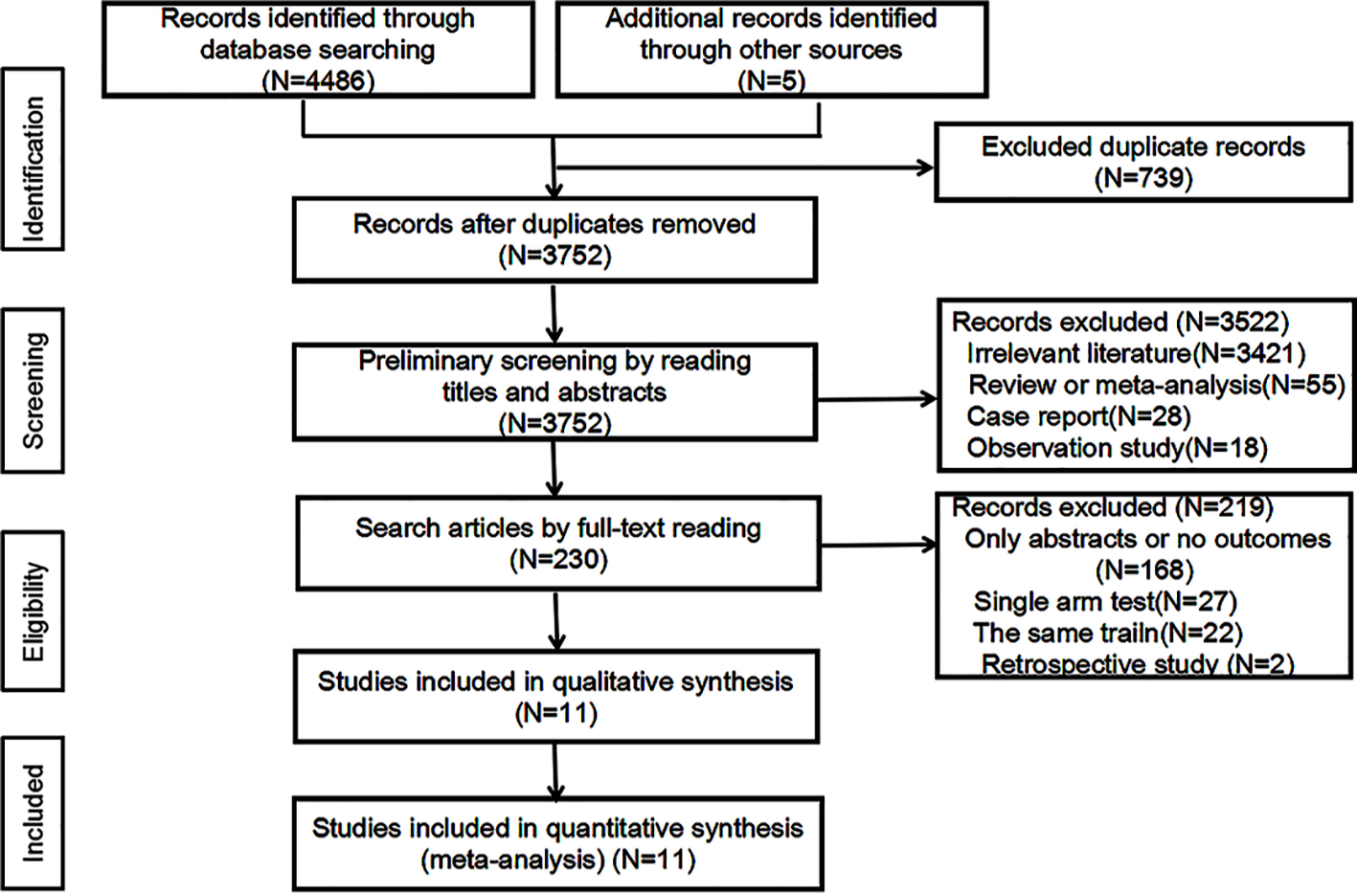
Flow chart of included study.

**Figure 2:**
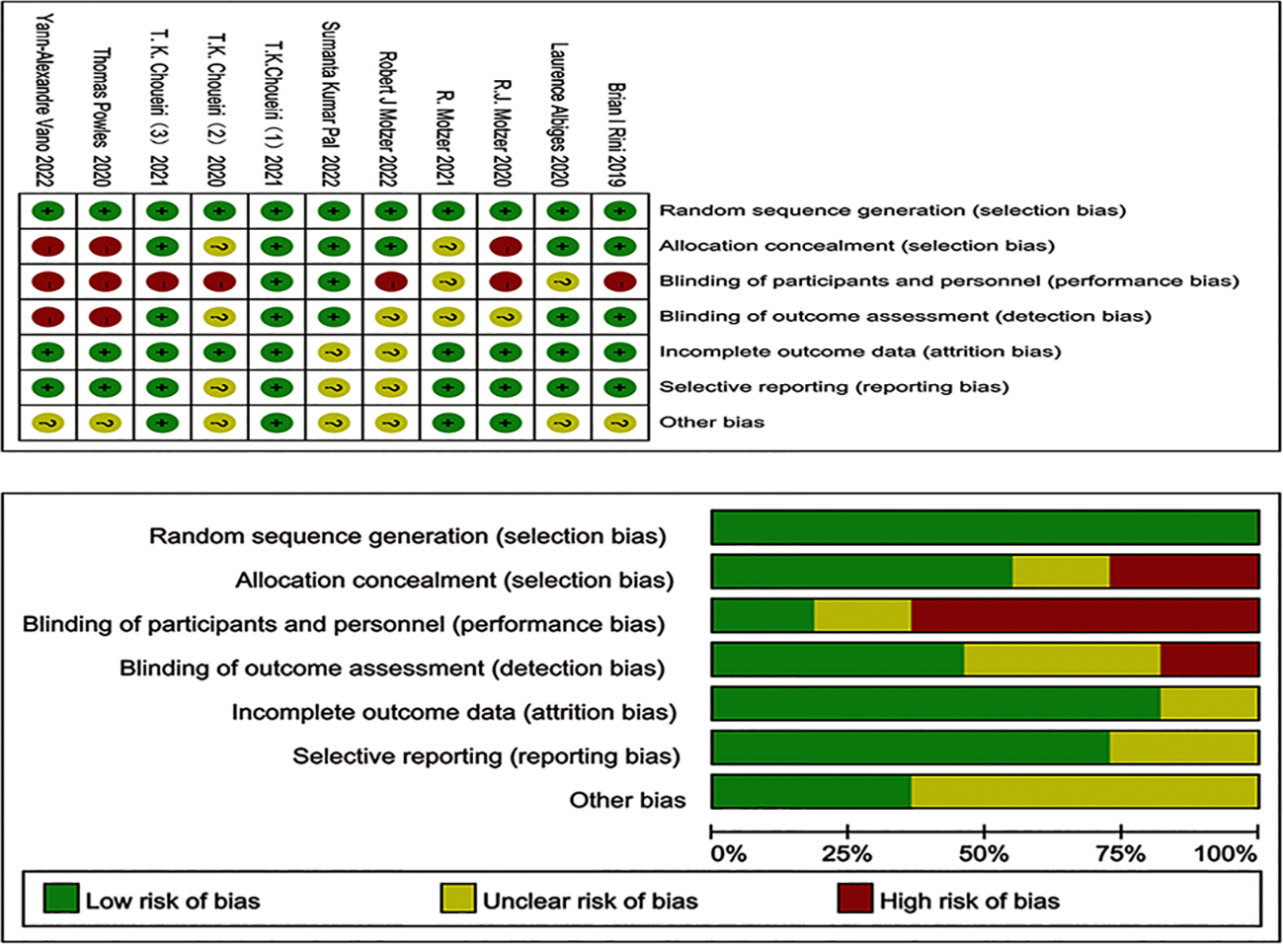
Risk bias of studies included.

A total of 4491 relevant papers from the preliminary search were identified. After excluding duplicate literature, 11 research articles were included for meta-analysis by analyzing the titles, abstracts, and full text. A flow chart corresponding to this process is provided below (**Figure 1**). A total of 7895 study subjects were enrolled, with 3936 cases in the experimental group and 3959 cases in the control group. The corresponding characteristics are shown in **Table 1**, and bias risk evaluation is shown in **Figure 2**.

**Table 1:** Basic information of included studies.

Included studies	Design	Sample number(n)		Intervention study		Outcome indicators
		Test group	Control group	Test group	Control group	
Sumanta Kumar Pal 2022 (9)	RCT	390(287/103)	388(278/110)	Atezolizumab plus bevacizumab	placebo	①⑦
T.K.Choueiri 2021(1) (10)	RCT	496(374/149)	498(359/139)	Pembrolizumab	placebo	①⑦
R.J.Motzer 2020 (11)	RCT	410(315/95)	411(304/107)	Nivolumab	Everolimus	①②③④⑤⑥⑦
Laurence Albiges 2020 (12)	RCT	550(413/137)	546(395/151)	Nivolumab plus ipilimumab	sunitinib	①②③④⑤⑥⑦
Thomas Powles 2020 (13)	RCT	432(308/124)	429(320/109)	Pembrolizumab plusAxitinib	sunitinib	①②③④⑤⑥⑦
T.K.Choueiri 2020(2) (14)	RCT	442	444	avelumab plus axitinib	sunitinib	①②③④⑤⑥
R.Motzer 2021 (15)	RCT	355(255/100)	357(275/82)	Pembrolizumab plus Lenvatinib	Sunitinib	①②③④⑤⑥⑦
Robert J Motzer 2022 (16)	RCT	323(249/74)	328(232/96)	Nivolumab plus cabozantinib	Sunitinib	①②③④⑤⑥⑦
Brian I Rini 2019 (17)	RCT	454(317/137)	461(352/109)	Atezolizumab plus bevacizumab	sunitinib	①②③④⑤⑥⑦
T. K. Choueiri 2021(3) (18)	RCT	47(35/12)	61(52/9)	avelumab plus axitinib	sunitinib	①②③④⑤⑥
Yann-Alexandre Vano 2022 (19)	RCT	37(33/4)	36(25/11)	Nivolumab plus ipilimumab	VEGFR-TKI	②③④⑤⑥⑦

①Overall survival ②Progression free survival ③Objective response rate ④Partial response ⑤Complete response⑥Disease control rate⑦Adverse effects incidence rate.

#### 3.1.1 Overall survival

**Figure 3:**
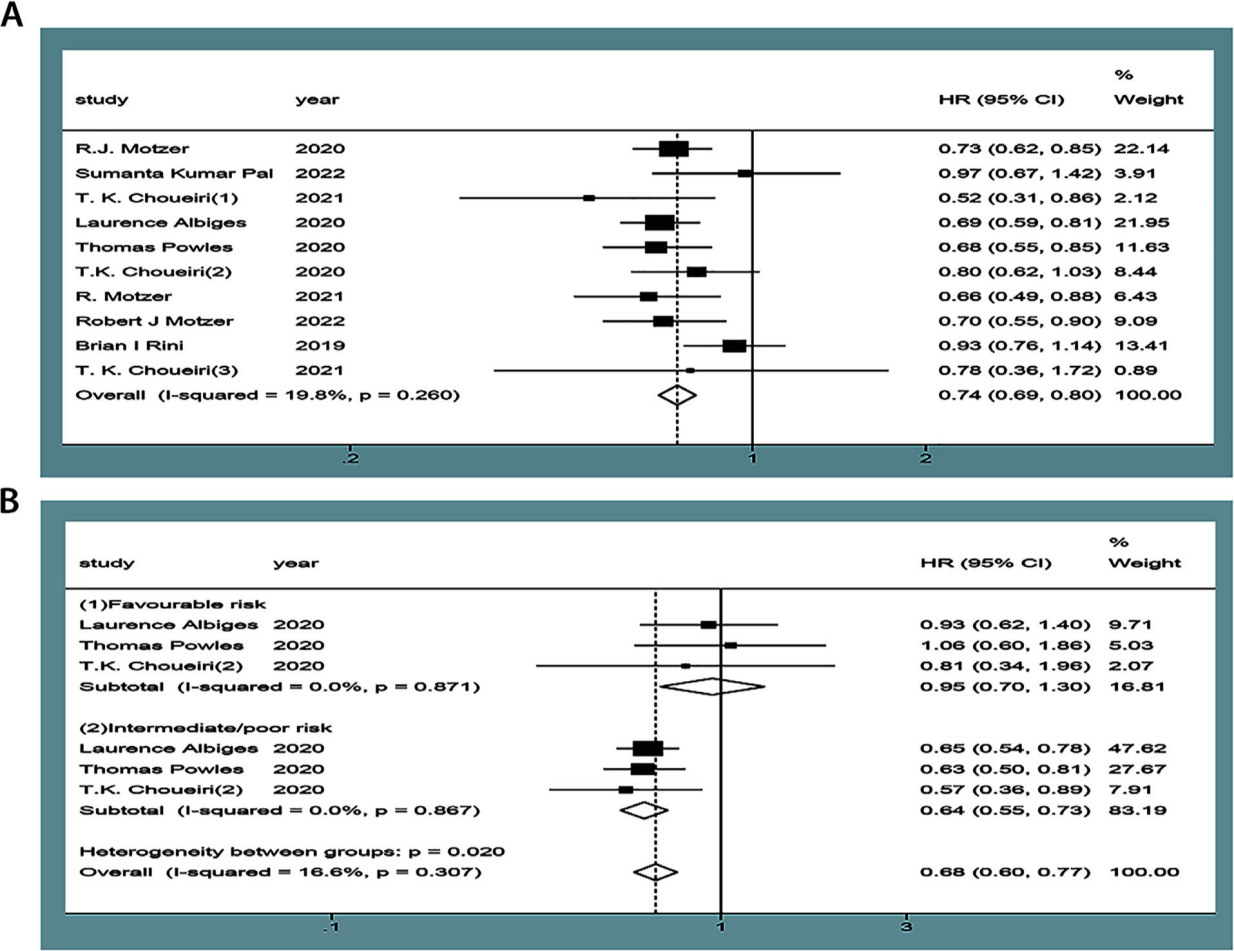
Forest plots of OS **(A)**. Forest plot of pooled OS basing on subgroup analyses **(B)**.

Ten articles reported OS (9–18), of which three (12–14) were used for subgroup analyses based on the risk score of the International Combined Database for Metastatic Renal Cell Carcinoma (IMDC). There was no statistical heterogeneity between the studies (*P*=0.26, *I^2^ =* 19.8% or *P*=0.31, *I^2^ =* 16.6%) with a random-effect model. The results showed that the OS of the experimental group in the treatment of mRCC or aRCC was significantly better than that of the control group, followed by the combined effect size (HR=0.74, 95%CI: 0.68~0.80, *P*<0.00001). Subgroup analysis showed that the experimental group could improve the IMDC score of the medium/high-risk group (HR=0.69, 95%CI: 0.55~0.73, *P*<0.00001), and the differences were statistically significant, suggesting that the single or combined use of PD-1/PD-L1 inhibitors could significantly improve OS in patients with mRCC or aRCC (**Figure 3**).

#### 3.1.2 Progression-free survival

**Figure 4:**
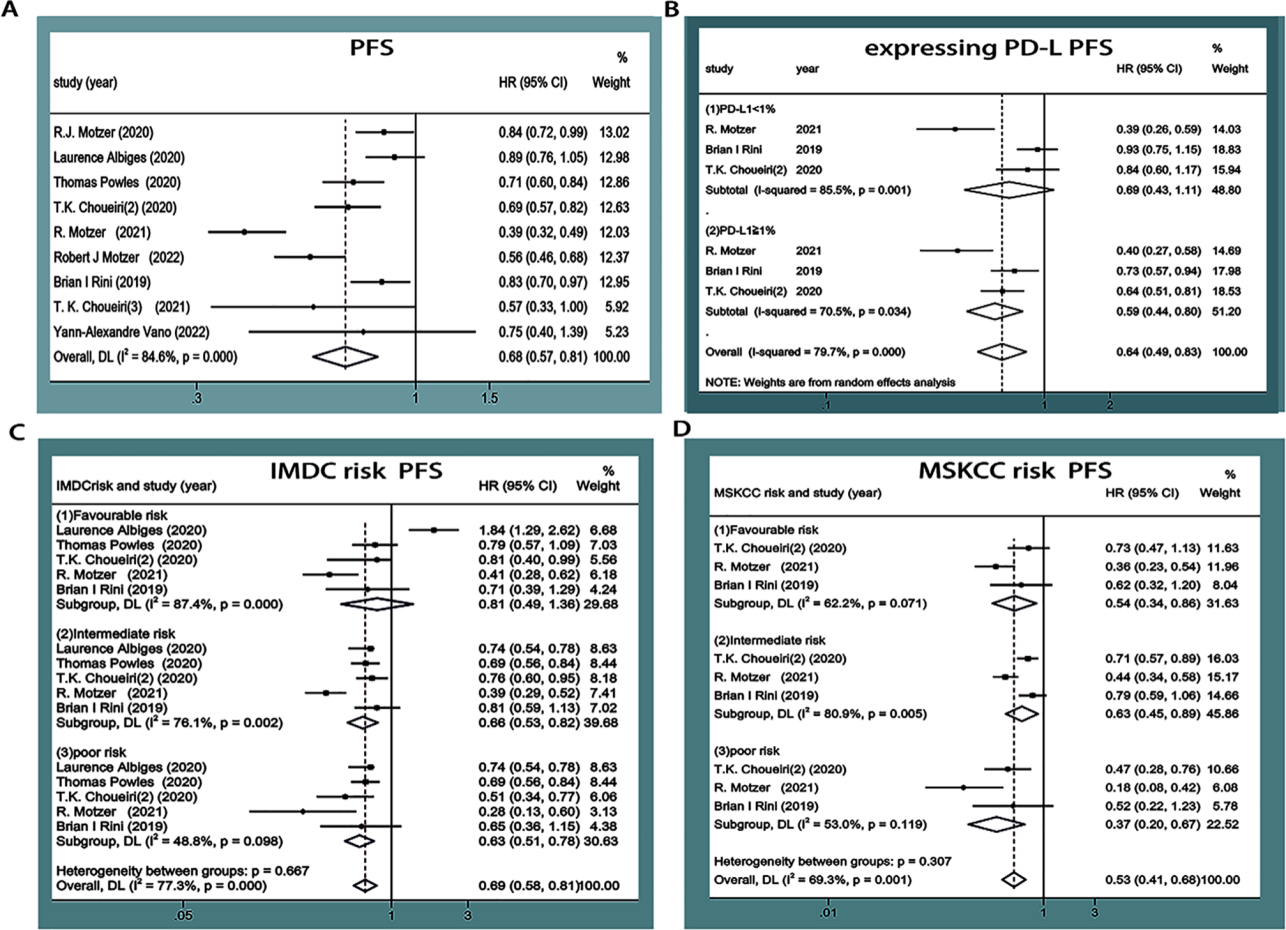
Forest plots of PFS **(A)**. Forest plot of pooled PFS basing on expressing PD-L1 subgroup analyses **(B)**. Forest plot of pooled PFS basing on IMDC risk subgroup analyses **(C)**. Forest plot of pooled PFS basing on MSKCC risk subgroup analyses **(D)**.

PFS was reported in nine articles (11–19), among which three articles (14, 15, 17) underwent subgroup analyses based on PD-L1 expression, four (12, 13, 15, 17) underwent subgroup analyses based on IMDC score, and three (14, 15, 17) underwent subgroup analyses based on the Memorial Sloan Kettering Cancer Center (MSKCC) score. Statistical heterogeneity was observed between the studies (*P*<0.00001, *I^2^ =* 84.6%; *P*<0.00001, *I^2^ =* 79.7%; *P<*0.00001, *I^2^ =* 77.3%; *P*=0.001, *I^2^ =* 69.3%). Random-effects model analysis showed that the PFS of mRCC or aRCC in the experimental group was significantly better than that of the control group, followed by the combined effect size (HR=0.68, 95%CI: 0.57~0.81, *P*<0.0001). Subgroup analysis showed that the PFS of different subgroup types was improved in the experimental group, PD-L1≥1% (HR=0.59, 95%CI: 0.44~0.80, *P*=0.001), IMDC score (medium-risk group, high-risk group) (HR=0.66, 95%CI: 0.53~0.82, *P*<0.0001; HR=0.63, 95%CI: 0.51~0.78, *P*<0.0001), and MSKCC score (medium-risk group, high-risk group) (HR=0.63, 95%CI: 0.45~0.89, *P*=0.008; HR=0.37, 95%CI: 0.20~0.67, *P*=0.001). The difference was statistically significant (*P*<0.05), suggesting that the PFS of patients with mRCC or aRCC was significantly prolonged by the single or combined use of PD-1/PD-L1 inhibitors (**Figure 4**).

#### 3.1.3 Objective response rate

**Figure 5:**
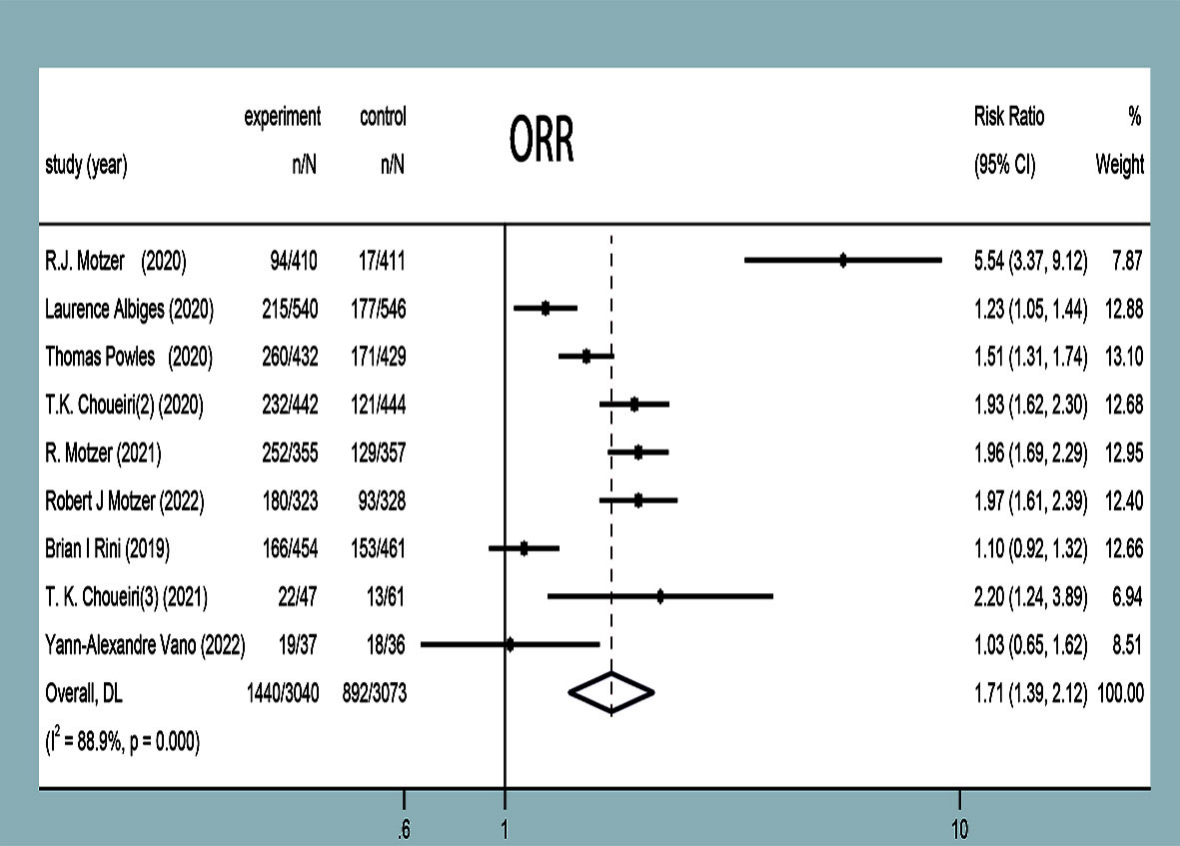
ORR forest plots in experimental group and control group.

ORR was reported in nine studies (11–19), and statistical heterogeneity was observed between them (*P*<0.0001, *I^2^ =* 88.9%). Random-effects model analysis showed that the ORR of the experimental group was significantly higher than that in the control group in terms of treating mRCC or aRCC, and with the combined effect size (RR=1.71, 95%CI: 1.39~2.12, *P*<0.00001), the difference was statistically significant (**Figure 5**).

#### 3.1.4 Partial response rate

**Figure 6:**
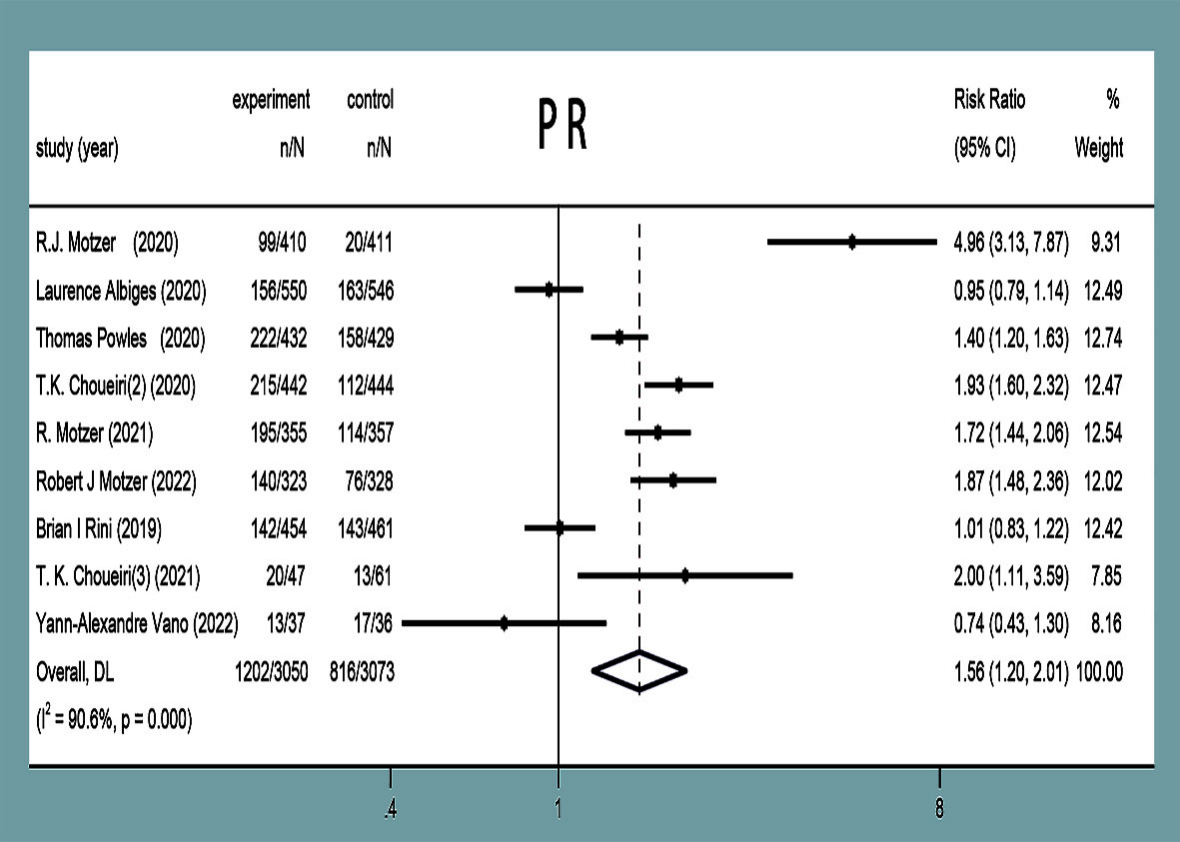
PR forest plots in experimental group and control group.

PR was reported in nine studies (11–19), with statistical heterogeneity observed between the studies (*P*<0.00001, *I^2^ =* 90.6%). Random-effects model analysis showed that the PR in the experimental group was higher than that in the control group in the treatment of mRCC or aRCC, and with the combined effect size (RR=1.56, 95%CI: 1.20~2.01, *P*=0.001), the difference was statistically significant (**Figure 6**).

#### 3.1.5 Complete response rate

**Figure 7:**
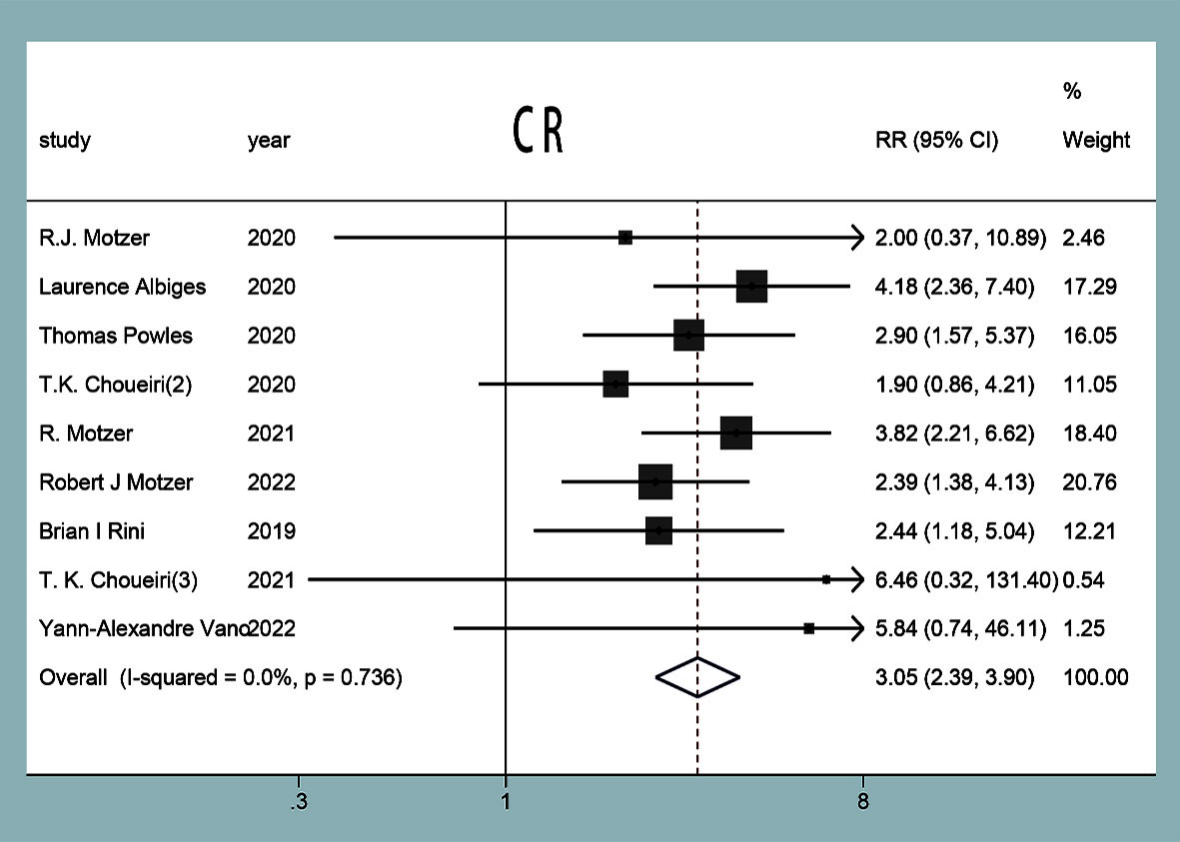
CR forest plots in experimental group and control group.

CR was reported in nine studies (11–19), and no statistical heterogeneity was observed between the studies (*P*=0.74, *I^2^ =* 0%). The results of the random-effect model showed that the CR of the experimental group was higher than that in the control group in the treatment of mRCC or aRCC, and with the combined effect size (RR=2.99 95%CI: 2.34~3.83, *P*<0.0001), the difference was statistically significant (**Figure 7**).

#### 3.1.6 Disease control rate

**Figure 8:**
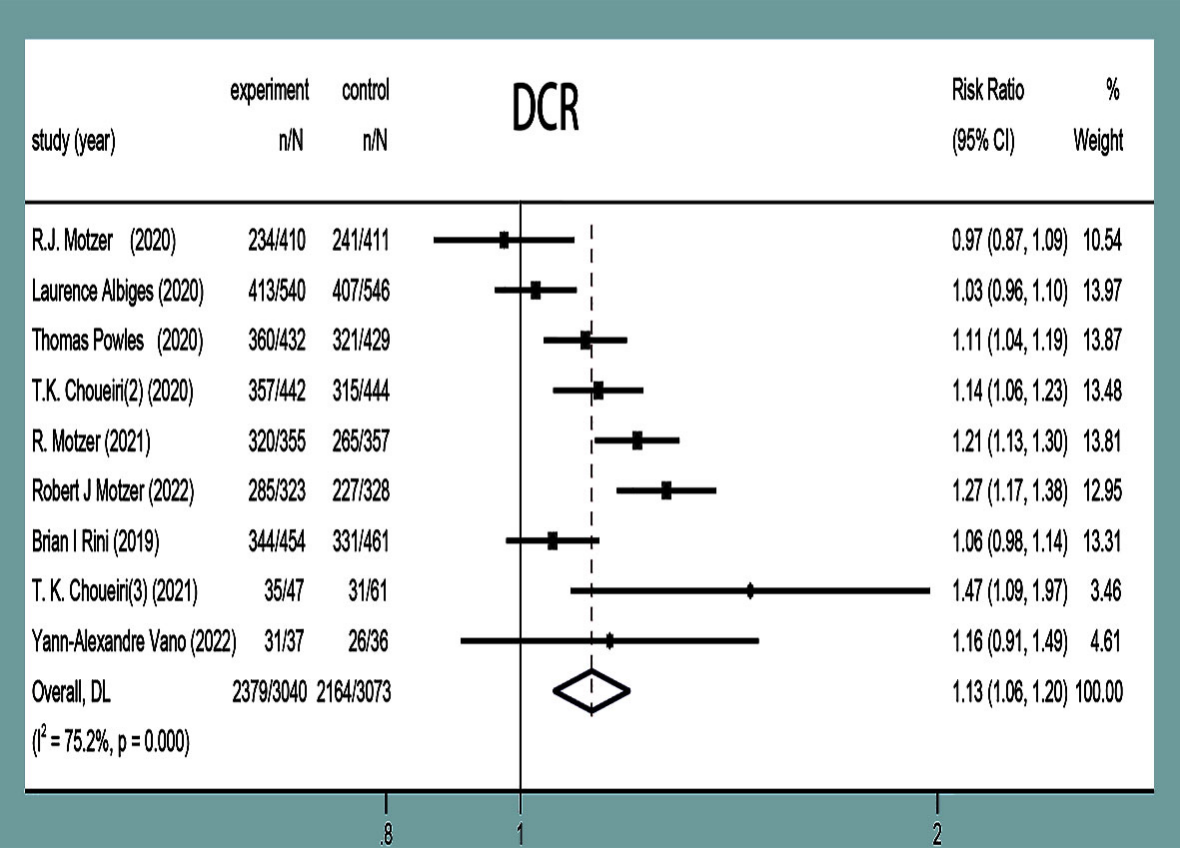
DCR forest plots in experimental group and control group.

DCR was reported in nine studies (11–19), and there was statistical heterogeneity between the studies (*P*<0.0001, *I^2^ =* 75.2%). Random-effects model analysis showed that the DCR in the experimental group was higher than that in the control group in the treatment of mRCC or aRCC, and with the combined effect size (RR=1.13, 95%CI: 1.06~1.20, *P*<0.0001), the difference was statistically significant (**Figure 8**).

#### 3.1.7 Adverse reactions

**Figure 9:**
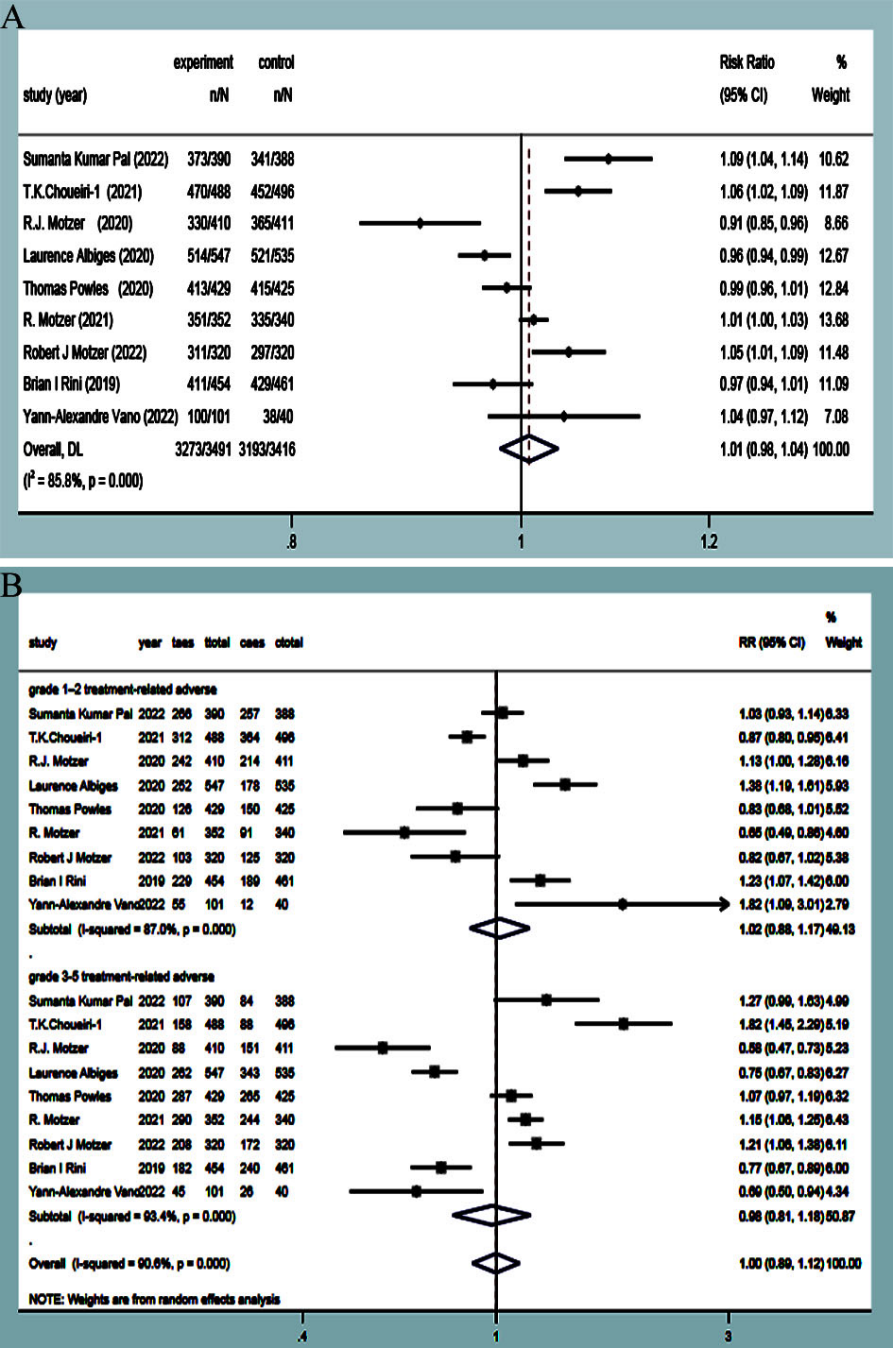
AES **(A)** and subgroup **(B)** forest plots in experimental group and control group.

AEs were reported in eleven studies (9–13, 15–17, 19), with statistical heterogeneity observed between the studies (*P*<0.00001, *I^2^ =* 85.8%). Random-effects model analysis revealed that there was no significant difference in AEs between the experimental group and the control group (RR=1.01, 95%CI: 0.98~1.04, *P*=0.598)(**Figure 9A**). Subgroup analysis showed that there were no significant differences in the incidence of stage I~II adverse reactions and III~V adverse reactions (RR=1.02, 95%CI: 0.88~1.17, *P*=0.818; RR=0.98, 95%CI: 0.81~1.18, *P*=0.817) (**Figure 9B**). Considering that the heterogeneity in the literature could cause non-statistically significant differences of AEs, subgroup meta-analysis of common adverse reactions was carried out according to the respiratory, digestive, and other systems provided in the studies.

##### 3.1.7.1 Respiratory system

**Figure 10:**
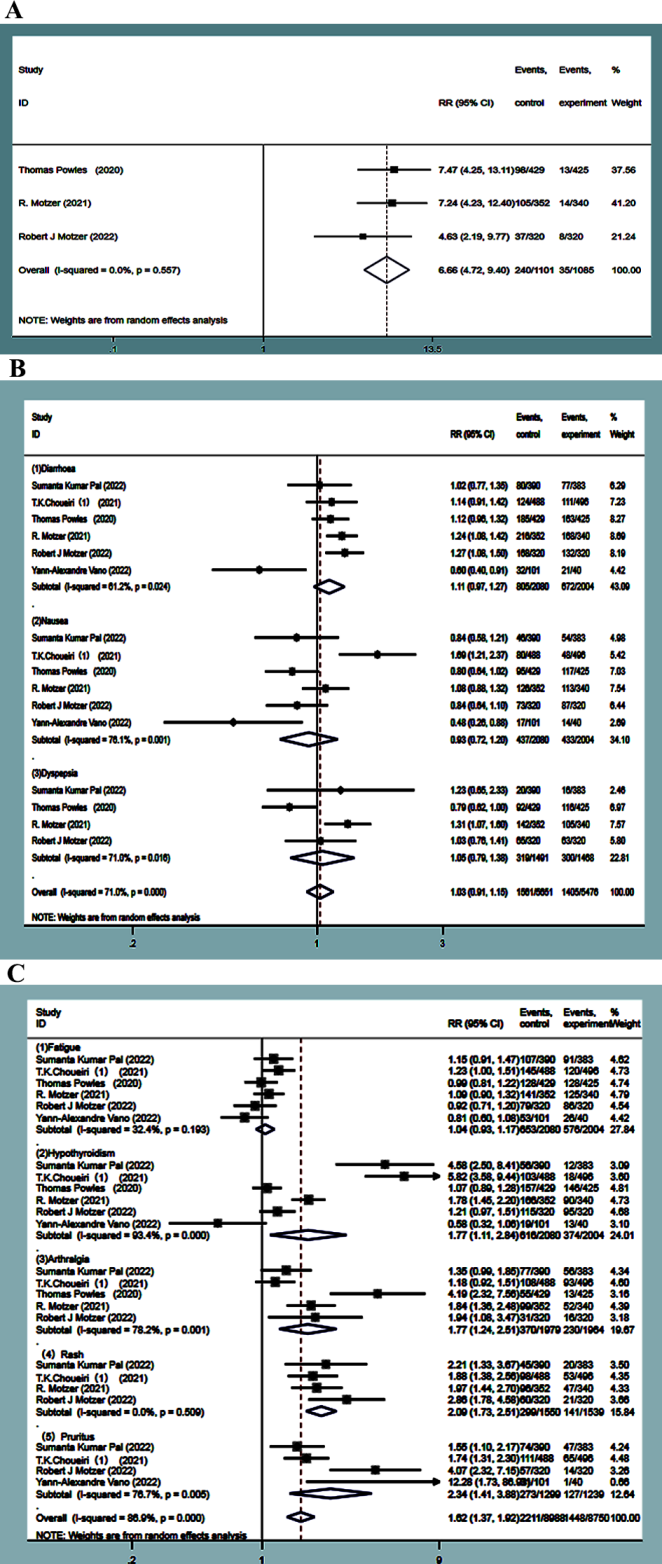
Dysphonia forest plots in experimental group and control group **(A)**. Adverse events of the digestive system forest plots in experimental group and control **(B)**. forest plots about adverse events of other system in experimental group and control **(C)**.

The incidence of dysphonia was higher in the experimental groups by about 21.8%. Dysphonia was reported in three studies (13, 15, 16). No statistical heterogeneity was observed between the studies (*P*=0.557, *I^2^ =* 0.0%), and the results of a random-effect model showed that the incidence of dysphonia in the experimental group was significantly higher than that in the control group. The difference was statistically significant (RR=6.66, 95%CI: 4.72~9.4, *P*<0.0001) (**Figure 10A**).

##### 3.1.7.2 Digestive system

The incidence of adverse reactions, such as diarrhea, nausea, and dyspepsia, was much higher in the experimental group, which was about 38.7%, 21%, and 21.3%. Six studies (9, 10, 13, 15, 16, 19) focused on diarrhea and nausea, and four studies (9, 13, 15, 16) reported dyspepsia. Statistical heterogeneity was observed between the studies *(P*=0.025, *I^2^ =* 61.1%; *P*=0.001, *I^2^ =* 76.1%; *P*=0.016, *I^2^ =* 71.0%), and random-effects model analysis showed that the incidence of adverse events, such as diarrhea, nausea, and dyspepsia, in the experimental group was not statistically significant compared to the control group (RR=1.11, 95%CI: 0.97~1.27, *P*=0.123; RR=0.93, 95%CI: 0.72~1.20, *P*=0.570; RR=1.05, 95%CI: 0.79~1.38, *P*=0.742) (**Figure 10B**).

##### 3.1.7.3 Other systems

The incidence of adverse reactions, such as fatigue, hypothyroidism, pruritus, rash, and arthralgia, was higher in the experimental group by 31.4%, 29.6%, 21.3%, 19.2%, and 18.7%, respectively. Six studies (9, 10, 13, 15, 16, 19) reported fatigue, while four (9, 10, 15, 16) reported rash. No statistical heterogeneity was observed between the studies (*P*=0.198, *I^2^ =* 31.7%; *P*=0.511, *I^2^ =* 0.00%). The random-effect model results showed that the incidence of fatigue had no statistical significance in the experimental group as compared with the control group (RR=1.04, 95%CI: 0.93~1.17, *P*=0.471), while the incidence of rash in the experimental group was significantly higher than that in the control group, followed by combined effect size (RR=2.09, 95%CI: 1.73~2.51, *P*<0.0001). Six studies (9, 10, 13, 15, 16, 19) studied hypothyroidism, five (9, 10, 13, 15, 16) reported on arthralgia, and four (9, 10, 16, 19) reported on pruritus, with statistical heterogeneity observed between them (*P*<0.0001, *I^2^ =* 90.3%; *P*=0.001, *I^2^ =* 77.9%; *P*=0.006, *I^2^ =* 75.7%). The random-effects model analysis indicated that the incidence of adverse reactions, such as hypothyroidism, arthralgia, and pruritus, in the experimental group was significantly higher than that in the control group. The difference was statistically significant (RR=1.77, 95%CI:1.12~2.84, *P*=0.015; RR=1.77, 95%CI: 1.24~2.51, *P*=0.001; RR=2.34, 95%CI:1.41~3.88, *P*=0.001) (**Figure 10C**).

### 3.2 Sensitivity analysis

**Figure 11:**
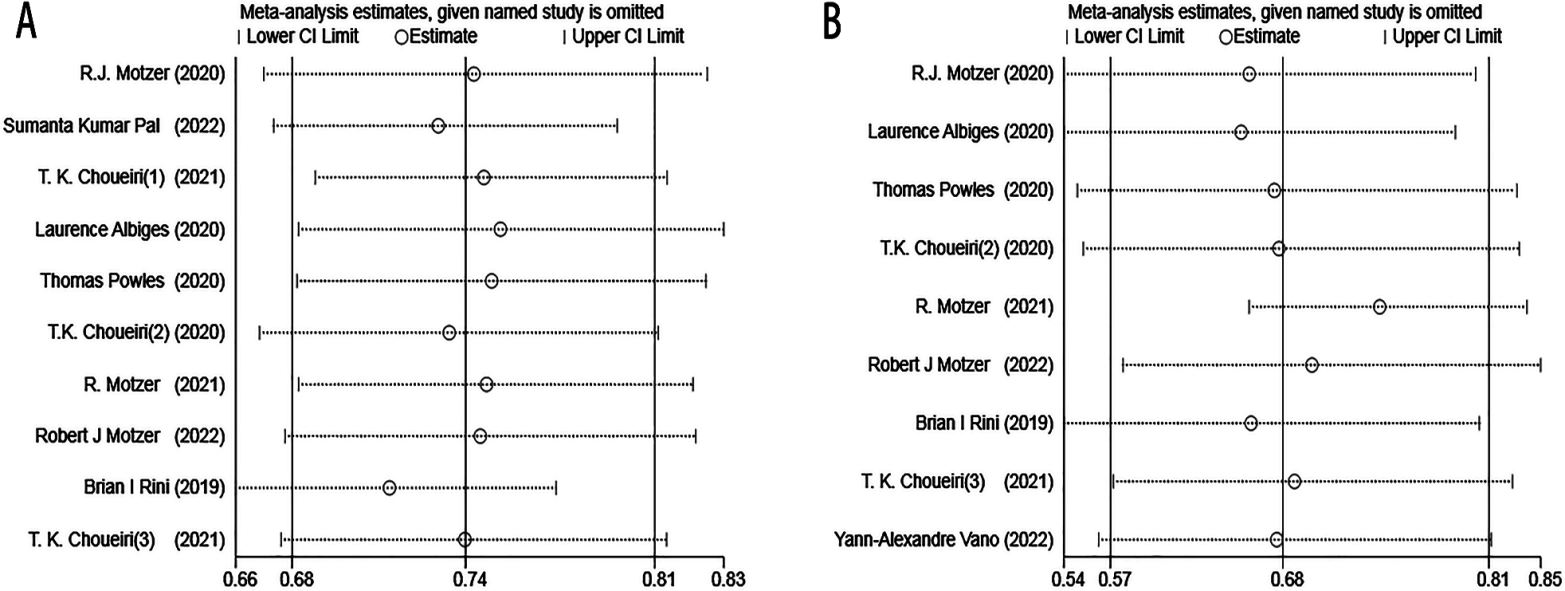
OS sensitivity analysis **(A)**. PFS sensitivity analysis **(B)**.

Sensitivity analysis was performed using the trim and filling method to assess the potential impact of each included study on combined HR. The results revealed the negligible stability of the comparison results, suggesting that the meta-analysis is reliable (**Figure 11**).

### 3.3 Assessment of the risks of bias

**Figure 12:**
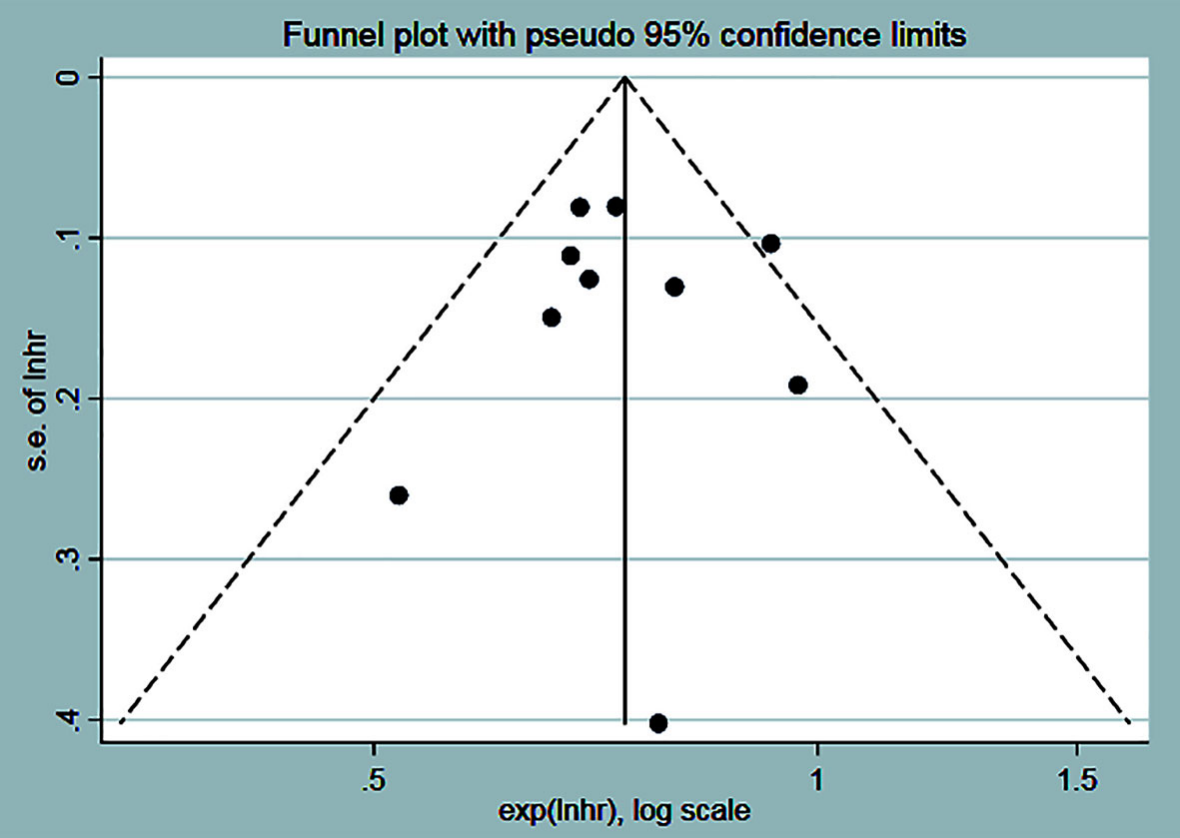
Scatterplots of 10 articles with OS.

The funnel plot and Egger test method were used to analyze the publication bias of OS in the experimental group and the control group. The funnel plot showed that the distribution of each study was symmetrical (**Figure 12**), and Egger’s test indicated that there was a low possibility of publication bias (*P*=0.987) (**Table 2**).

**Table 2:** Egger’s test.

Std_Eff	Coef	SE	t	P>|t|	95%CI
slope	-0.299	0.119	-2.51	0.036	(-0.574,-0.246)
bias	-0.016	0.994	-0.02	0.987	(-2.309,2.276)

## 4 Discussion

A total of 11 high-quality foreign randomized controlled trials in phase II and III were included in the systematical analysis and evaluation of the clinical efficacy and safety of PD-1/PD-L1 inhibitors alone or in combination in the treatment of mRCC or aRCC, with OS, PFS, ORR, PR, CR, DCR, and AEs as the outcome indicators. Firstly, in terms of total clinical efficacy, it was found that the experimental group was significantly better than the control group in OS, PFS, ORR, PR, CR, and DCR, indicating that the single or combined use of PD-1/PD-L1 inhibitors can produce better clinical benefits in the treatment of mRCC or aRCC, especially in terms of prolonging the OS and PFS of patients. Secondly, with regards to safety, no statistical significance was observed between the experimental group and the control group in terms of total AEs, stage I~II adverse reactions, or stage III~V adverse reactions, indicating that the PD-1/PD-L1 inhibitors alone or in combination did not increase the incidence of adverse reactions in patients, indicative of their safety. Although the incidence of adverse reactions, such as dysphonia, rash, hypothyroidism, arthralgia, and itching, in the experimental group was significantly higher than that in the control group, these reactions were deemed controllable. Therefore, in clinical treatment, these risks should be identified and subsequently avoided when treating mRCC or aRCC when using PD-1/PD-L1 inhibitors. It has been shown that the safety and tolerability of first-line VEGF inhibitors, including sunitinib, have been demonstrated in the treatment of renal cell carcinoma. And in this study, 7 of them were controlled studies with sunitinib group, and through my study, we found that PD-1/PD-L1 monotherapy or combination therapy group significantly prolonged OS, PFS, and improved ORR compared with sunitinib group, and significantly attenuated the incidence of AEs associated with any level of treatment. There are 2 papers with controlled studies with placebo group, and through my study, I found that there was no increase in grade 3 or higher treatment-related adverse events in the PD-1/PD-L1 mono or combination therapy group compared to the placebo group, which would suggest that combining PD-1/PD-L1 targeted agents is promising and does not increase drug toxicity. Thus, PD-1/PD-L1 monotherapy or combination therapy showed a very good safety profile in patients with advanced renal cell carcinoma. RCC, the twelfth most common cancer in the world (20), has no characteristic clinical features at the early stage. As a result, as many as 25% to 75% of patients develop distant metastases by the time of diagnosis, missing lifesaving opportunities for surgery. In addition, the prognosis of patients with mRCC or aRCC is always poor, with a five-year survival rate of less than 10% (21). Even with radical renal cancer resection, postoperative recurrence rates range from 10% in low-risk patients to 68% in high-risk patients (22). The widespread application of targeted therapy in cancer has greatly improved prognosis and prolonged the OS and PFS of kidney cancer patients (23). However, the efficacy of targeted drugs like sunitinib as a first-line medicine is not satisfied in the treatment of some mRCCs and aRCCs due to drug resistance (24). PD-1/PD-L1 serves as a common stimulator of the T cell immune response. When the PD-1/PD-L1 signaling pathway is activated, it can reduce damage to surrounding tissues by the immune response (25). In the tumor microenvironment, signaling overactivation can inhibit the proliferation and activation or apoptosis of CD4 and CD8T cells, thereby promoting the negative regulation of immunity and inhibiting the immune effect of T cells and lymphocytes. This will help tumor cells escape the surveillance of somatic immune response cells, eventually forming immune tolerance. It could mediate the immune escape of tumor cells and promote cellular growth and proliferation without immune prevention and control (26, 27). According to the guidelines of the European Society of Medical Oncology and the European Association of Urology (28), for clear cell renal cell carcinoma patients with medium- or high-risk operation, PD-1/PD-L1 inhibitors represented by pembrolizumab can be used to assist in postoperative radical resection or adjuvant immunotherapy with mRCC. This has also been shown to achieve good survival benefits in multicenter, randomized clinical trials of CheckMate 025, CheckMate 214, and KEYNOTE-426 (29, 30).

In this study, after systematically assessing and analyzing the seven outcome indicators of OS, PFS, ORR, PR, CR, DCR, and AEs, it was not difficult to find that, in terms of efficacy and safety, the OS, PFS, ORR, PR, CR, DCR, and AEs of the PD-1/PD-L1 inhibitor group, alone or in combination with the PD-1/PD-L1 inhibitor group, had a significantly better clinical efficacy and safety in the treatment of mRCC or aRCC than the control group. Significant heterogeneity was found in the analysis of PFS, and subgroup analyses were performed by PD-L1 expression, IMDC score, and the Memorial Sloan Kettering Cancer Center (MSKCC) score. As a result, it was found that the heterogeneity still existed, which would suggest that the heterogeneity of PFS may come from factors such as race, follow-up time, and sample size, which had less impact on our study in terms of the effectiveness of PFS. On the contrary, in terms of the heterogeneity of AEs, by classifying common adverse reactions by disease system and performing subgroup analyses, the results showed that the heterogeneity of adverse reactions, such as hairiness, rash, and dysphonia, was significantly reduced, and the burden of safety was significantly reduced compared with that of the control group, further indicating that the existence of heterogeneity of AEs could be eliminated by systematic subgroup analyses. It has been shown that racial differences exist in the clinical efficacy of cancer patients receiving targeted therapies in different regions, and the efficiency of patients with PD-1/PD-L1 inhibitors may vary depending on geographic location, which will likely be another important reason for the presence of heterogeneity. At the same time, we cannot ignore the following reasons for the existence of heterogeneity: (i) the sample size, population distribution, ethnicity, and tumor type of the included studies were quite different; (ii) there were differences in the traditional first-line chemotherapy regimen of patients receiving PD-1/PD-L1 treatment; (iii) most of the literature failed to specifically describe the random allocation method and allocation concealment, causing some non-blind results to the experimenter, investigator and experimental; (iv) in the experiment, the patient withdrew and lost to follow-up due to serious adverse reactions or other reasons. Therefore, we will continue to update the data and collect more high-quality clinical trials, especially in terms of medication regimen, duration of follow-up, type of disease, and ethnicity, to conduct a more detailed analysis to validate the data presented in this paper. But this study has the advantages of a long search time, the most comprehensive outcome indicators, the most recent data from literature studies and a large sample size compared with similar published literature, thus being a more objective and comprehensive outcome indicator for the analysis. At the same time, subgroup analyses were conducted for each outcome indicator, eliminating heterogeneity between studies and providing more objective and reliable guidance for clinical treatment.

In summary, the single or combined use of PD-1/PD-L1 inhibitors shows significant clinical benefits and safety in treating mRCC or aRCC. However, due to the limited number of articles, more high-quality, multicenter randomized clinical studies are needed to gain more comprehensive and sufficient medical rationales for treating mRCC or aRCC.
